# Editorial: Using digital solutions for Brief Interventions in Alcohol, Tobacco, other drug use, and gambling: From the present to the future

**DOI:** 10.3389/fdgth.2023.1123942

**Published:** 2023-02-06

**Authors:** Hugo López-Pelayo, Elsa Caballeria, Antoni Gual, Silvia Matrai, Michael P. Schaub

**Affiliations:** ^1^ Unitat de Conductes Addictives, Hospital Clínic Barcelona, Barcelona, Spain; ^2^ Addictions Research Group, Institut d’Investigacions Biomèdiques August Pi I Sunyer, Barcelona, Spain; ^3^ Swiss Research Institute of Public Health and Addiction ISGF, University of Zurich, Zurich, Switzerland

**Keywords:** brief intervention (BI), alcohol, drugs, digital health, gambling, cannabis, tobacco

**Editorial on the Research Topic** Using digital solutions for Brief Interventions in Alcohol, Tobacco, other drug use, and gambling: From the present to the future

Approximately 269 million people used drugs at least once last year, and 36 million have a substance use disorder worldwide (1). Alcohol is responsible for three million deaths every year, and 13.5% of deaths in young people (20–39-year-olds) are attributable to alcohol (2). Over one billion people use tobacco, a substance that is the cause of death for eight million people every year (3). Between 0.1% and 6% of the world's population have a gambling disorder (4). Brief interventions (BI) are effective and cost-effective in reducing alcohol and other drug use (5). Nevertheless, the implementation of brief interventions is still scarce in clinical practice (5). Given the increasing coverage of smartphones, wearables, and other connected devices worldwide, digital health solutions have the potential to scale up the delivery of brief interventions and overcome the main barriers to their implementation (e.g., the risk of upsetting the patient, privacy and confidentiality concerns, access to rural areas, and vulnerable populations).

The implementation of BIs has long been a relevant concern. In 2014, digitally-driven modalities were proposed to contribute to overcoming barriers to the large-scale and sustained implementation of BIs for alcohol and other drug use (6).

In 2018, the Special Interest Group on digital interventions of the International Network on Brief Interventions for Alcohol & Other Drugs (e-INEBRIA SIG) proposed a roadmap for this area of research, covering the following topics: (1) the evaluation of effective implementation modalities, (2) diversification and cultural sensitivity, (3) the accommodation of new technology, and (4) intervention quality and safety management (7).

This Research Topic aims to advance the development of digital solutions to improve the efficacy and facilitate the implementation of brief interventions in the areas of alcohol, tobacco, cannabis, prescribed or illicit drug use, and gambling. “Digital solutions” refers to electronic and communication technologies used to support, complement, or substitute face-to-face BIs (either the online BI alone or a mixed intervention).

This special issue includes five manuscripts authored by 26 renowned researchers, covering diverse areas such as the development of an artificial-intelligence-powered human avatar for BIs to prevent alcohol misuse (Pahola (R) and the effectiveness of a mobile intervention to reduce alcohol use in young people. Pahola (R) is freely available in Spanish, Portuguese, and English on the Pan American Health Organization (PAHO) website. In less than a year, 236,000 sessions were registered on Pahola's landing pages, mostly through mobile devices Monteiro et al. Preliminary evidence on Pahola has shown the potential of this new approach, which may become a game changer for brief interventions. Schulte et al. compared a mobile phone intervention with an educational website control group in an RCT in young people and found no differences between them. One possible explanation is that the control condition (website) involved an overly active intervention.

Blended learning focused on substance misuse for students is a promising approach to overcoming implementation barriers in this sensitive population Griffin et al. Targeting impulsivity in a brief cognitive training task seems to have enormous potential as an element of brief interventions, yet further research is needed to attain conclusive evidence Hwang et al. The long-term impact of brief interventions on alcohol misuse remains an open question, in the response to which, Baumann and colleagues take an important step forward by studying the long-term effects of BI according to the trajectories of alcohol use in the general population. Despite the lack of insight into the long-term effects of brief interventions on both low- and high-risk patterns, personalised feedback regarding the alcohol use of individuals appears to be an active component of brief interventions for those with low drinking patterns Baumann et al.

While this Research Topic could not cover the broad roadmap proposed by e-INEBRIA SIG, it represents the real progress that has been achieved in the field. As the world and its population are facing major challenges (COVID-19 pandemic, climate-related natural disasters, armed conflicts, etc.) leading to socioeconomic crises, new challenges and opportunities are emerging in the field of digital mental health, including alcohol and other drug use. In this context, it is essential to empower people to self-manage their well-being and strengthen healthcare systems as well as their actors (frontline professionals, managers, policymakers, etc.) to deal with substance misuse and mental health problems. Despite the effectiveness and cost-effectiveness of most digital mental interventions, including those for substance misuse, there is a mismatch between the supply of solutions and people's needs. The integration of digital solutions into existing care practices and infrastructures (e.g., software) can be improved, and large-scale approaches are scarce, with few publicly funded digital approaches having been put in place. In summary, beyond the roadmap, further challenges can be identified in relation to the implementation of digital health interventions, most notably:

1) How can digital solutions address unmet prevention and treatment needs in substance misuse and other high-risk lifestyle behaviours?
2) What is the role of socioeconomic determinants of the digital divide and what can be done to overcome their effects, and in particular, prevent growing health inequalities?
3) What can research do to facilitate the integration of digital solutions into sustainable, large-scale approaches?
4) What are the context-specific priorities in diverse societies and cultures regarding digital interventions for substance misuse? Can researchers flexibly adapt to contextual needs at an appropriate pace?
5) How can digital solutions be embedded in the health system so that they become practical tools shared by patients and health professionals?

Probably the most relevant question is how to adapt training to the new generation of researchers who have already been born in a digitalised world and are eager to benefit from the enormous research achievements in this field to date. Training and networking programs for researchers need to be updated with content on how to monitor and forecast rapidly changing digital trends, develop assessment strategies for digital solutions that are resistant to technology obsolescence, incorporate the environmental impact of both the digital solution and the targeted lifestyle behaviour as a core part of any research, and take an intersectional approach to vulnerabilities and the digital divide when designing solutions.
